# Development of a Next-Generation Cooling Channel Technology with High Cooling Efficiency by Roughing Cooling Channels Using a Combination of Laser Machining and Embossing Techniques

**DOI:** 10.3390/mi16020225

**Published:** 2025-02-16

**Authors:** Chil-Chyuan Kuo, Geng-Feng Lin, Armaan Farooqui, Song-Hua Huang, Shih-Feng Tseng

**Affiliations:** 1 Department of Mechanical Engineering, Ming Chi University of Technology No. 84, Gungjuan Road, New Taipei City 24301, Taiwan; 2 Research Center for Intelligent Medical Devices, Ming Chi University of Technology, No. 84, Gungjuan Road, New Taipei City 24301, Taiwan; 3 Department of Mechanical Engineering, Chang Gung University, No. 259, Wenhua 1st Road, Guishan District, Taoyuan City 33302, Taiwan; 4 Center for Reliability Engineering, Ming Chi University of Technology, No. 84, Gungjuan Road, Taishan District, New Taipei City 24301, Taiwan; 5 Department of Mechanical Engineering, Chhattisgarh Swami Vivekanand Technical University, Bhilai 491107, Chhattisgarh, India; 6 Li-Yin Technology Co., Ltd., No. 37, Lane 151, Section 1, Zhongxing Road, Wugu District, New Taipei City 248012, Taiwan; 7 Department of Mechanical Engineering, National Taipei University of Technology, No. 1, Section 3, Zhongxiao E. Road, Da’an District, Taipei City 106344, Taiwan

**Keywords:** aluminum-filled epoxy resin molds, surface roughness, heat dissipation, cooling efficiency

## Abstract

This study investigates the development of a rapid wax injection tooling with enhanced heat dissipation performance using aluminum-filled epoxy resin molds and cooling channel roughening technology. Experimental evaluations were conducted on cooling channels with eleven surface roughness variations, revealing that a maximum roughness of 71.9 µm achieved an 81.48% improvement in cooling efficiency compared to smooth channels. The optimal coolant discharge rate was determined to be 2 L/min. The heat dissipation time for wax patterns was significantly reduced, enabling a cooling time reduction of approximately 12 s per product. For a production scale of 100,000 units, this equates to a time savings of about 13 days. Empirical equations were established for estimating heat dissipation time and pressure drop, with a high coefficient of determination. This research provides a valuable contribution to the mold and dies manufacturing industry, offering practical solutions for sustainable and efficient production processes.

## 1. Introduction

Aluminum-filled epoxy resin is classified as a composite material. Epoxy resins [1], known for their thermosetting nature, are extensively utilized across various industries owing to their superior mechanical strength, adhesive qualities, and chemical resistance. When aluminum particles [2] are incorporated into the epoxy matrix, they form an aluminum-filled epoxy resin. The addition of aluminum fillers enhances the thermal, electrical, and mechanical properties of this composite material, while still preserving the essential characteristics of the epoxy resin. The thermal conductivity of normal epoxy resin is 0.3 W/m*K, while the aluminum-filled epoxy resin used in this study is 1.071 W/m*K. Rapid tooling uses rapid prototyping and digital manufacturing technologies to create molds, and the global market for this technology grows steadily. The global rapid prototyping market size is estimated at USD 3.33 billion in 2024, is expected to grow to USD 4.01 billion in 2025, and is predicted to reach around USD 21.47 billion by 2034, expanding at a CAGR of 20.49% between 2024 and 2034 [3]. Future advancements in 3D printing and digital manufacturing, along with the adoption of Industry 4.0, further boost the rapid tooling market. Wax injection molding is a process used to create investment-casting molds. First, molten wax goes into a metal mold to form a wax pattern that replicates the final product. Next, the wax patterns are assembled onto a wax tree and coated with ceramic slurry to build a ceramic shell. The wax then melts out, leaving a hollow ceramic mold. Finally, molten metal pours into the mold, and the ceramic shell breaks away to reveal the finished metal parts. This method produces detailed and precise metal components for industries like aerospace, automotive, and jewelry. Cooling time is crucial in wax injection molding or plastic injection molding, affecting product quality, dimensional accuracy, as well as material properties [4]. Proper cooling ensures that the wax solidifies uniformly, preventing defects like warping and sink marks, while maintaining precise dimensions. Optimizing cooling time improves production efficiency and makes mold release easier, balancing quality and cost.

To reduce cooling time during wax injection molding, mold design can be optimized by incorporating cooling channels for better heat dissipation [5]. However, this approach introduces maintenance challenges, risks of leakage, and can weaken the mold’s structural integrity and lifespan. Careful design, thorough evaluation, and regular maintenance are crucial to balancing these factors for optimal mold performance. Injection pressure and speed can be adjusted to ensure uniform filling and faster cooling. Advanced cooling systems, such as water-cooling or air-cooling, can be implemented to efficiently remove heat from the mold. However, optimizing mold design with cooling channels increases mold complexity and cost. Raising the mold temperature can cause uneven wax solidification and defects, and it also raises energy consumption. Using materials with high thermal conductivity is more expensive and may wear out quickly, needing more maintenance. Adjusting injection pressure and speed can cause more wear on equipment, higher energy use, and potential defects like flash. Implementing advanced cooling systems is costly and complex, requiring extra space and maintenance [6]. Kariminejad et al. [7] utilized selective laser sintering to fabricate sensor-integrated mold inserts with both conformal and bent channels for the positioning of adjustable thermocouples. Their findings revealed that conformal cooling can reduce production cycle times by around 50% and improve the quality of the final product compared to traditional cooling methods. Additionally, the adjustable thermocouples embedded in the bent channels provided precise measurements of mold temperature and coolant effects at two specific spots within the 3D-printed inserts [8]. Bianchi et al. [9] developed a novel thermal management system that leverages regional mold temperatures and Peltier modules to independently regulate the temperature of different cavity features based on their thickness. By integrating finite element analysis with particle swarm optimization, this system optimizes regional temperature profiles over time, ensuring steady cooling rates across the molded parts. The operation of this system was contrasted to fixed peripheral mold temperature and quick heat cycle molding practices, which intend for consistent temperatures across the mold cavity surface. The outcomes demonstrated that the presented approach, which emphasizes local temperature control and consistent cooling rates, allows for the parallel solidification of attributes with substantial differences in surface-to-volume ratios. This method offers enhanced dimensional accuracy and reduces differential shrinkage, thereby improving the injection molding process’s ability to produce ceramic components with varying wall thicknesses. Mascher et al. [10] investigated the extension of flow path lengths in molds designed for thin-walled packaging or thick-walled technical components with extensive flow paths. The study examined a mold surface processed through electrical discharge machining. By modifying operation settings, the molding compound, and surface roughness, the study assessed the impact of mold surface structure on the attainable flow path length. A flow meander mold was used for this analysis. The results indicated that surface roughness positively influences flow path length, though this effect is material-dependent and relatively minor compared to other factors like melting or mold temperature.

Understanding the core technology of cooling channel design for wax injection molds is crucial. The unique design of the heat dissipation channel increases the contact area, enhancing cooling efficiency by allowing the coolant to absorb more heat. Research in fluid mechanics, supported by preliminary experiments, demonstrates that micro-structured or rough mold surfaces can lower flow resistance and shear rate while extending flow path lengths [11,12,13,14,15,16]. Laser ablation processes can be used to introduce microstructures into the mold surface [17,18]. Laser ablation provides extremely precise control over material removal, allowing for the creation of intricate microstructures and complex designs. It reduces thermal damage to surrounding areas by minimizing heat-affected zones. This method is versatile and can be applied to various materials such as metals, ceramics, polymers, and composites. As a non-contact technique, it avoids mechanical stress and wear on both the material and the equipment. Furthermore, laser ablation generates fewer residues and pollutants, ensuring a cleaner working environment. Especially, embossing can also be employed to introduce textures onto the mold surface [19,20,21]. This technique involves pressing a patterned mold or stamp into the mold material to imprint the desired surface textures. Embossing is well-suited for creating repetitive patterns and can achieve high precision, depending on the quality of the mold and the embossing process. This study uses fiber lasers and embossing to create varying roughness for the cooling channel surface, then employs rapid tooling to produce molds for wax injection molding. An investigation was conducted to analyze the cooling efficiency of water channels featuring various surface textures, utilizing a low-pressure wax injection molding technique.

## 2. Experimental Details

**Figure 1:**
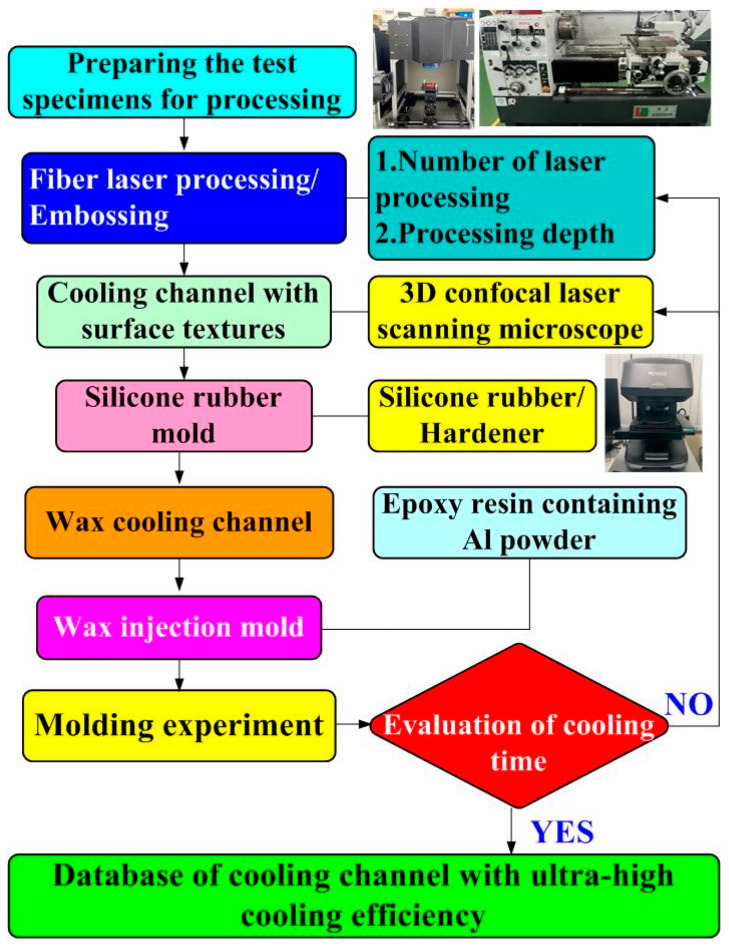
Flowchart depicting the investigation framework for this study.

**Figure 2:**
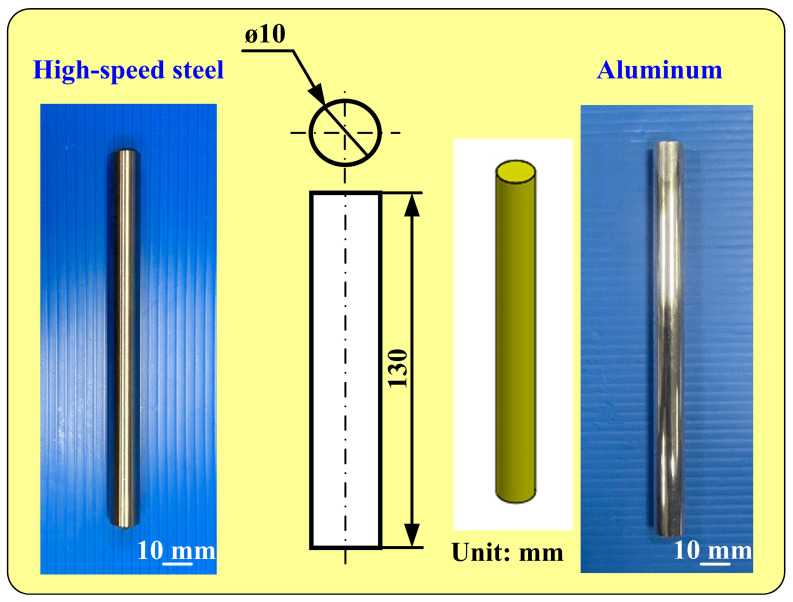
Two specimens used to create cooling channels with varying surface roughnesses.

The investigation framework for this study is illustrated in the flowchart presented in Figure 1. To create a cooling channel with high cooling efficiency, the first thing to do is to process the sample with fiber laser or embossing technology to create the surface texture of the cooling channel. Machining parameters, such as the number of laser processes and the processing depth, are also recorded. The machined sample surface acquires a specific microstructural texture to form a functional cooling surface. The silicone base and hardener are mixed in proportion to create a silicone mold that is used to replicate the texture of the sample. A silicone mold is used to create a wax cooling channel that serves as an intermediary model for the final mold. Then, an epoxy resin containing aluminum powder is poured into a wax mold to form a metal resin cooling channel mold. The coolant mold was used for low-pressure injection molding, and the cooling efficiency was evaluated in an experiment. In this study, the definition of cooling time is based on the time it takes for the wax temperature in the mold to drop from the starting temperature to the set target temperature. The target temperature during the cooling process is set at 30 °C, the critical temperature range required for the wax to change from liquid to solid. Specifically, we look at the temperature data measured by a single thermocouple inside the mold, and when the measured temperature steadily drops below the solidification temperature of the wax and tends to stabilize, the cooling process is considered complete. This usually reflects that the wax inside the mold has fully cured. Finally, the cooling time required for the mold to cool the injection molded product is tested, and the cooling effect of the mold is verified by experimental data. This study selects two materials as specimens, i.e., aluminum and high-speed steel. Figure 2 shows the two specimens used to create cooling channels with different surface roughnesses. Each processed rod has a length of 120 mm and a diameter of 10 mm. A lathe embosses aluminum rods to create cooling channels with three types of high surface roughness. This research employs laser and embossing machining methods to modify the surface of cooling channels, achieving various levels of surface roughness. Rapid tooling technology is then utilized to fabricate an injection molding mold. Embossing machining for aluminum rods was carried out using a turning machine. A fiber laser machine was used to process high-speed steel rods for creating specific surface textures of cooling channels. The focusing lens features a 125 mm focal length, a 50 × 50 mm^2^ scanning area, a top rate of scan of 5000 mm/s, and a spot diameter of 40 µm at the focus. The fiber laser operates at a wavelength of 1064 nm, with a peak average power of 30 W, a maximum pulse rate of 1 MHz, a beam quality factor of 1.5, and a pulse duration of 4 ns. A fiber laser processes high-speed steel rods to produce eight types of surface roughness, from low to medium. In total, 11 different surface roughness levels, ranging from low to high, are produced on the cooling channel surfaces. In this study, a fiber laser processes eight different surface roughnesses on high-speed steel rods with a power of 28 W, a processing speed of 30 mm/s, and a processing spacing of 0.05 mm. The fiber laser processes these eight cooling channels on high-speed steel rods with different surface roughnesses, applying 1, 3, 5, 7, 9, 11, 13, and 15 passes to each. The parameters for embossing are a rotation speed of 330 rpm and a feed rate of 0.01 mm/s. An embossing tool produces eight cooling channels with varying surface roughness at the following three embossing depths: 0.2 mm, 0.6 mm, and 1.2 mm. The resulting surface roughnesses are investigated with a three-dimensional confocal laser scanning microscope. Sz (maximum height) surface roughness quantifies the vertical distance between the tallest peak and the deepest valley within a defined surface area [22]. A surface profilometer and other analysis instruments trace the surface profile to determine this parameter. It plays an essential role in industries like manufacturing and engineering, where surface finish impacts performance in sealing, friction, and wear applications. Organizations like ISO and ASME provide standards for measuring and reporting Sz. In summary, Sz offers valuable insights into surface topography, ensuring surfaces meet required specifications.

Table 1 presents the parameters that directly affect the cooling performance. The total heat that needs to be removed for complete cooling of the wax mold is 15.2 kJ, calculated based on the wax’s mass, specific heat capacity, and temperature difference from 85 °C to 30 °C. While the surface roughness of the cooling channel plays a role in heat dissipation by affecting turbulence and convective heat transfer, it is only one of many factors influencing cooling time. The thermal conductivity of the wax (0.211 W/m·K), mold thickness (4 cm), and aluminum particle content (6:4 aluminum-to-epoxy ratio) also significantly impact heat transfer efficiency. Furthermore, the interaction between contact resistance, mold geometry, and coolant flow behavior influences overall cooling performance. Given these dependencies, the empirical equations derived in this study are valid only under the specified conditions, and their application to other molds should account for all relevant thermal factors.

**Figure 3:**
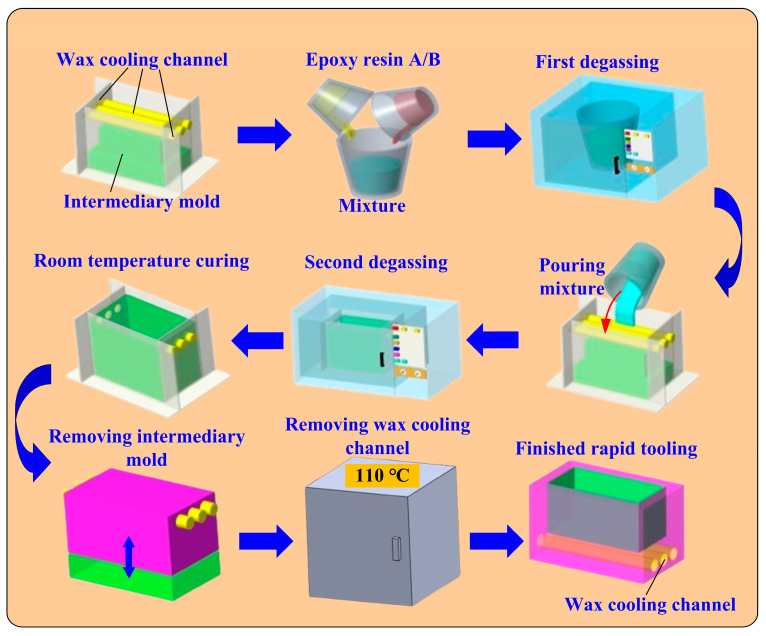
Detailed fabrication process of rapid tooling with ultra-high cooling performance.

Figure 3 shows the detailed fabrication process of rapid tooling with ultra-high cooling performance. The detailed fabrication process of rapid tooling with ultra-high cooling efficiency includes several key steps. First, processed specimens are used to create silicone rubber molds using silicone rubber, which can completely transfer the roughness of the surfaces of the cooling channel. Next, silicone rubber molds are used to create the wax-cooling water channel, which can be removed by heating the mold produced by rapid tooling. The intermediary mold and the wax cooling channel are installed in the mold frame. A mixture for fabricating a rapid tooling is prepared. The mixture undergoes two rounds of degassing using a vacuum degassing machine before being poured inside the mold frame. After solidifying the rapid mold at room temperature, it is separated from the intermediate mold, and the wax cooling channel is removed using heat in the oven. This process achieves rapid molding with ultra-high cooling efficiency. To create rapid tooling with epoxy resin containing 70% aluminum powder, a precise computer-aided design model is initially created. Then, a master pattern is prepared in a mold box and a release agent is applied. The rapid tooling measures 100 mm in length, 100 mm in width, and 60 mm in height. The epoxy resin, hardener, and aluminum fillers are thoroughly mixed, ensuring no air bubbles are present. This mixture is then poured into the mold box, with vibration applied to help it settle, and it is allowed to cure. After curing, the epoxy is demolded, inspected for defects, and the mold is trimmed and finished as needed. Finally, the mold must be tested to confirm it meets the required specifications and is ready for production. The aluminum powder possesses an approximate particle size of 50 µm. The purity of the aluminum powder is about 98%. The matrix materials for fabricating rapid tooling with ultra-high cooling performance are mixed from a curing agent and a base compound of the epoxy resin.

The heat dissipation efficiency of the water channels with these varying surface textures is investigated through a series of wax injection molding experiments using an apparatus. Heat dissipation efficiency is a measure of how effectively a system dissipates heat. For cooling systems, the efficiency can be expressed as:

η = (T_hot,in_ − T_hot,out_)/(T_hot,in_ − T_cold,in_) (1)

**Figure 4:**
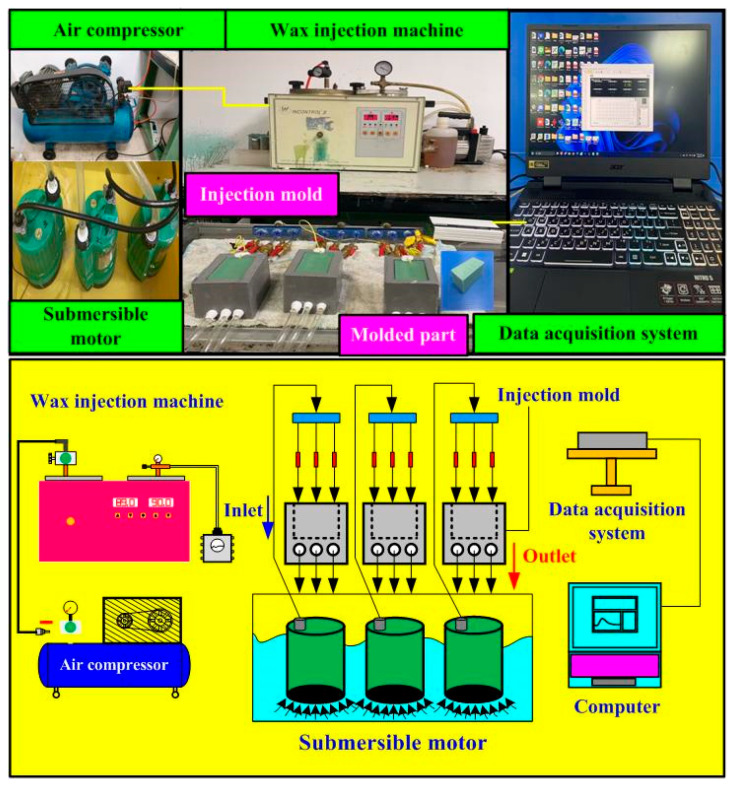
Homemade experimental apparatus to evaluate the cooling efficiency of the wax pattern.

The cooling efficiency of the water channels with varying surface textures was investigated through wax injection molding experiments using a homemade experimental apparatus, as shown in Figure 4. This setup features a thermoelectric cooler connected to a coolant reservoir, three K-type thermocouples with an accuracy of ±1 °C, and a regulator for mold temperature integrated into the system. This system captures temperature data using a data acquisition system. The horizontal low-pressure wax injection mold receives molten wax at approximately 85–90 °C, while the mold cavity is set at a room temperature of approximately 27–30 °C. The Dye Tracing Method was used to inject a colorant into a fluid and observe the movement path of the dye to understand the flow pattern of the fluid. A high-resolution camera was used to take pictures of the flow experiments, and the distribution of streamlines was deduced with the theory of fluid mechanics.

## 3. Results and Discussion

**Figure 5:**
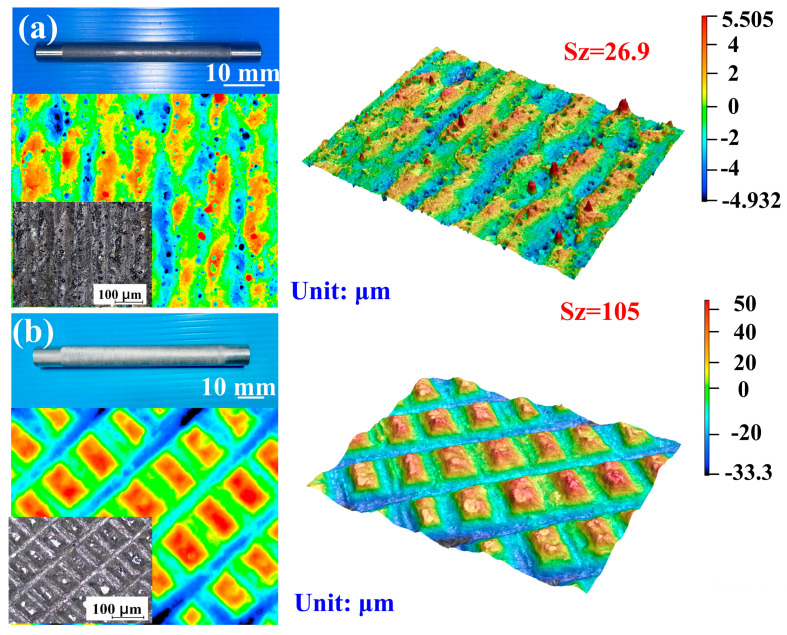
Surface texture of the test specimen after processing with (**a**) 15 laser passes or (**b**) an embossing depth of 0.6 mm.

Five repetitions of each experiment were performed. The surface roughness of the cooling channel is 2.4 µm, 3.2 µm, 4.1 µm, 4.9 µm, 12.2 µm, 16.5 µm, 19.5 µm, and 26.9 µm when the specimens are processed using a fiber laser 1, 3, 5, 7, 9, 11, 13 and 15 times, respectively. Furthermore, the roughness of the surface of the cooling channel measures 71.9 µm, 105 µm, and 274 µm when aluminum rods are processed with embossing depths of 0.2 mm, 0.6 mm, and 1.2 mm, respectively. Figure 5 illustrates the surface texture of the test specimen post-processing.

**Figure 6:**
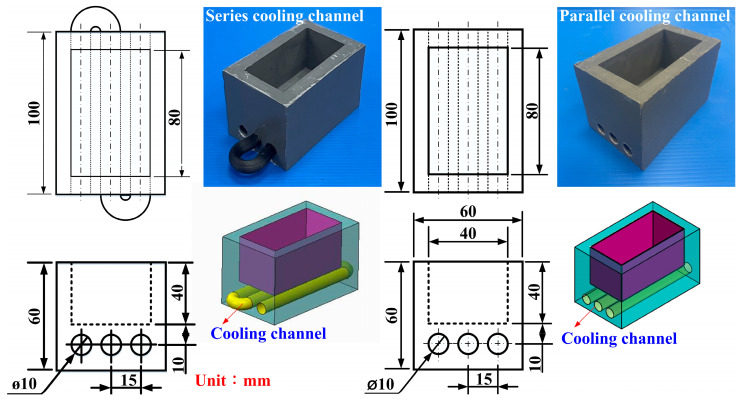
Rapid tooling with parallel or series cooling channels.

Parallel cooling channels promote uniform cooling and lower pressure loss by simultaneously distributing the coolant through multiple paths [23]. Series cooling channels, on the other hand, allow the coolant to absorb heat progressively. Still, the increasing coolant temperature reduces the temperature gradient over time, which can limit heat transfer efficiency. The choice between the two depends on whether uniform temperature control or higher local heat absorption is the primary goal. Figure 6 shows the rapid tooling with parallel or series cooling channels. The heat dissipation time, using parallel cooling channels with varying roughness of surfaces, is illustrated in Table 2. The measurement target temperature was set to 30 °C ± 0.5 °C to account for any truncation error. The cooling channel with surface roughness Sz 1.9 µm is the original surface roughness, meaning no processing is performed on the cooling channel surface. Table 3 shows the cooling time of wax patterns using a series of cooling channels with different surface roughnesses. Increasing the roughness of the surface of the cooling channel leads to the fastest heat dissipation time for the wax patterns.

**Figure 7:**
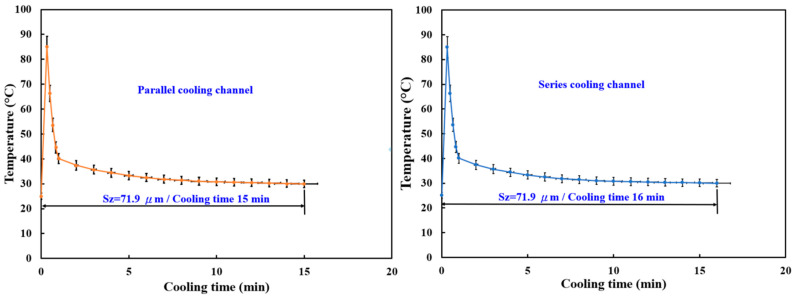
Relation between the wax pattern temperature and heat dissipation time of wax patterns with the fastest cooling times for the parallel and series cooling channels.

Figure 7 presents a comparative analysis of the cooling performance of parallel and series cooling channels, with a surface roughness (Sz) of 71.9 μm for both configurations. The results indicate that the parallel cooling channel achieves the fastest cooling time of 15 min, whereas the series cooling channel requires 16 min to reach the same temperature. The slightly faster cooling in the parallel configuration can be attributed to more uniform heat dissipation, as multiple channels simultaneously extract heat from different regions of the mold. In contrast, the series cooling channel exhibits a marginally longer cooling time due to the sequential heat transfer process, where the coolant must travel through the entire system before effectively reducing the mold temperature.

**Figure 8:**
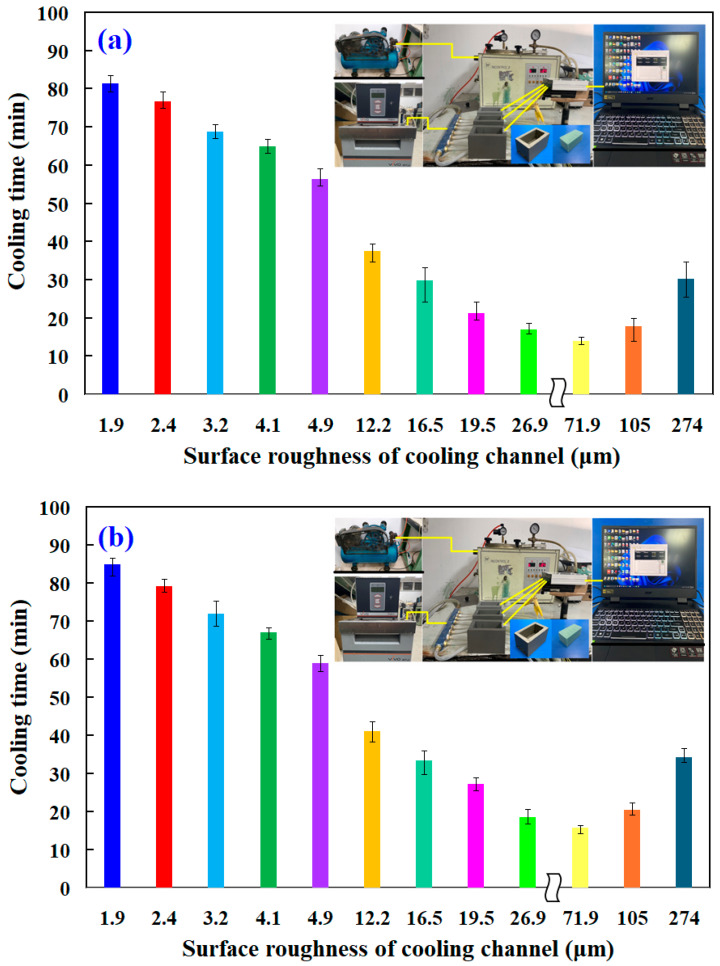
Heat dissipation time of wax patterns measured using rapid tooling connected in (**a**) parallel or (**b**) series cooling channels with different surface roughnesses.

Heat dissipation time is when molten wax in the rapid tooling solidifies and cools enough for secure ejection without distortion. Efficient heat dissipation is critical for developing high-quality wax patterns. Figure 8 shows the heat dissipation time of wax patterns measured using rapid tooling with parallel or series cooling channels with varying surface roughnesses. For the cooling channels connected in parallel, the heat dissipation time of the fabricated wax pattern (y) is determined by the maximum surface roughness (x) according to the equation y = 0.2015x^3^ − 3.2829x^2^ + 6.4581x + 76.615 with a correlation coefficient of 0.9928. For the cooling channels connected in series, the heat dissipation time (y) is determined by the maximum surface roughness (x) according to the equation y = 0.2022x^3^ − 3.2993x^2^ + 6.5816x + 79.457. The applicability of empirical equations is confined to the conditions of the experiment. In general, the maximum of the correlation coefficient is one. Note that this proposed predicted equation has a correlation coefficient of about 0.9919.

The cooling process of the wax mold was simulated using Moldex3D software, with a grid size of 1 mm to ensure accurate temperature distribution calculations. The ambient temperature and initial mold temperature were both set at 25 °C, while the initial wax temperature was 85 °C. The simulation continued until the wax temperature reached 30 °C. The cooling channels were modeled with varying surface roughness levels to analyze their influence on heat transfer performance. The cooling medium was water, which entered the channels at a constant temperature of 25 °C. The flow rate of water was set according to experimental conditions, ensuring a realistic representation of the cooling process. The convective heat transfer coefficient inside the channels was calculated using empirical correlations for turbulent water flow, which significantly enhances heat dissipation. The external surfaces of the mold were exposed to natural convection, with a heat transfer coefficient of 10 W/m^2^K, accounting for heat exchange with the surrounding air. Other parameters were manually set to match the actual experimental conditions, ensuring that the simulation accurately represented real-world behavior.

**Figure 9:**
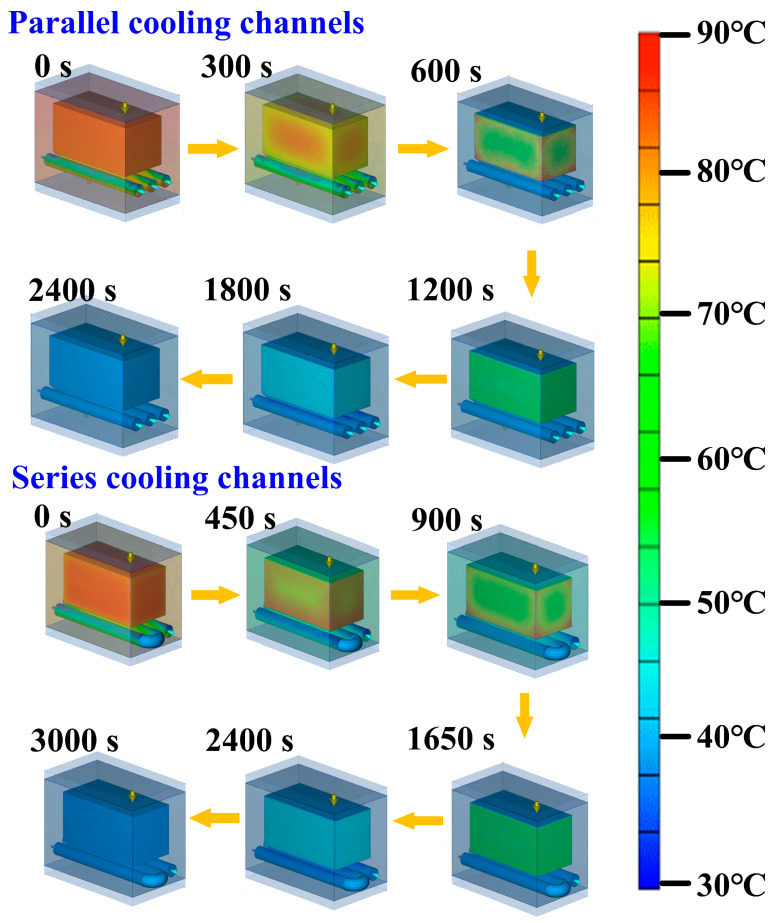
Cooling evolution of wax pattern fabricated by rapid tooling connected in parallel or series cooling channels.

Figure 9 illustrates the cooling progression of a wax pattern produced through rapid tooling, linked by parallel cooling channels. Three phenomena are observed for the cooling channels connected in parallel. First, the heat dissipation of the wax pattern is related to the cooling channel inside the rapid mold. Surface roughness is relevant. Second, increasing the surface roughness of the cooling channel results in the shortest cooling time for the wax patterns. This indicates that when rapid tooling, the increased surface roughness of the internal cooling channel provides the best heat dissipation efficiency. Third, the heat dissipation time of wax patterns using a cooling channel with surface roughness Sz 71.9 µm can be reduced by about 66 min compared to the original cooling channel. Therefore, the heat dissipation performance of the wax pattern can be increased by about 81.48%. For instance, considering a standard cooling time of 15 s for plastic injection molded products [24], this approach can save about 12 s per product. Extrapolated to a production run of 100,000 plastic injection molded products, this would equate to a total cooling time reduction of approximately 13 days. For the cooling channels connected in series, the heat dissipation time of wax patterns using the cooling channel with surface roughness Sz 71.9 µm can be reduced by about 69 min compared to the original cooling channel. Thus, the heat dissipation performance of the wax pattern can be increased by about 81.17%.

**Figure 10:**
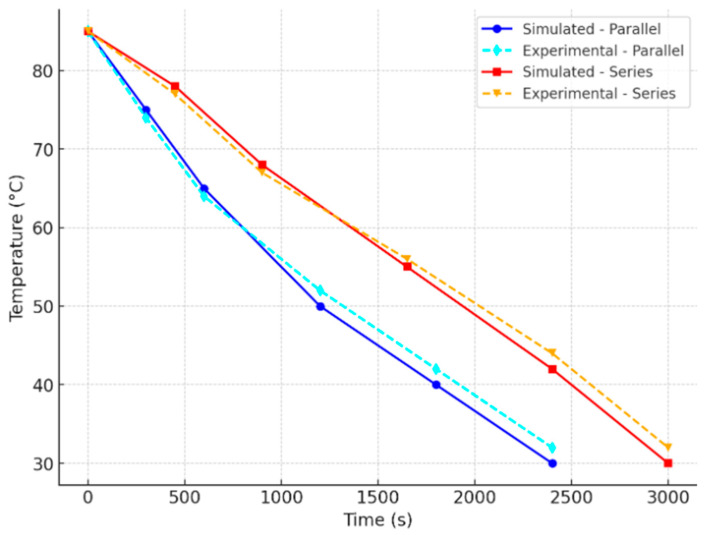
Comparison of experimental and simulation results of temperature variation.

To validate the accuracy of the simulation, a comparison was made with experimental temperature measurements taken at various probe locations. Figure 10 shows the evaluation of experimental data with simulation results. The results demonstrated a close correlation between the simulated and actual temperature profiles, confirming the reliability of the computational model. Discrepancies within a 5% margin of error were observed, which could be attributed to minor variations in experimental conditions such as flow turbulence and material inconsistencies.

**Figure 11:**
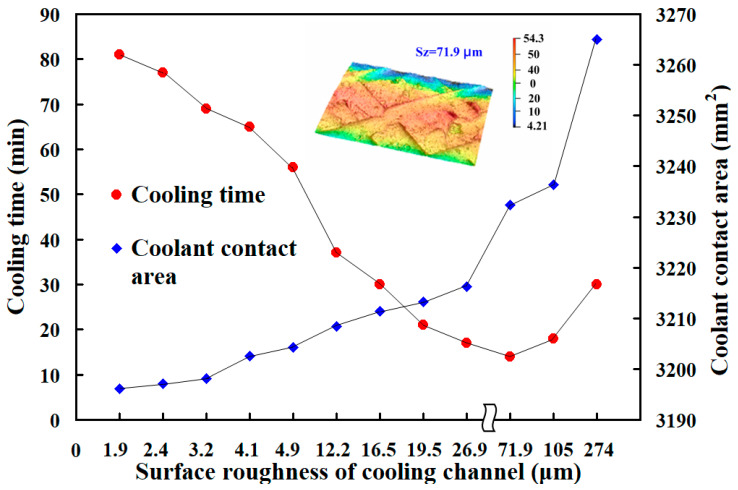
Relation between the cooling time and the coolant contact area with different surface roughnesses.

**Figure 12:**
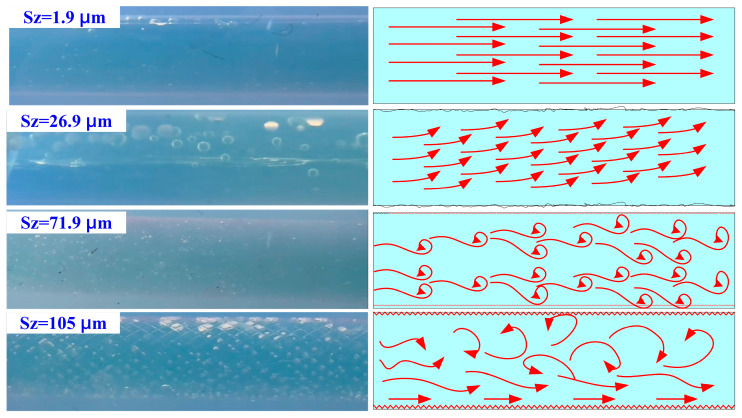
Graphic depiction of the flow characteristics of cooling channels with different surface roughnesses.

This study examines the influence of cooling channel surface roughness on cooling time and coolant contact area. The coolant contact area is the total area where the coolant is actually in contact with the surface of the inner wall of the cooling channel. The calculation should consider the effect of surface roughness on the actual contact area. The higher the surface roughness, the higher the degree of concave and convex microstructure, increasing the actual contact area. With the help of optical microscopy or 3D surface profiler measurements, the increase in the contact area under a rough surface can be quantified. Figure 11 shows the relation between the heat dissipation time and the coolant contact area with different surface roughnesses. Results indicate that the cooling times observed are approximately 81, 77, 69, 65, 56, 37, 30, 21, 17, 14, 18, and 30 min. The corresponding coolant contact areas are 3196 mm^2^, 3197 mm^2^, 3198 mm^2^, 3202 mm^2^, 3204 mm^2^, 3208 mm^2^, 3211 mm^2^, 3213 mm^2^, 3216 mm^2^, 3232 mm^2^, 3236 mm^2^, and 3265 mm^2^, respectively. The results indicate two significant findings. The shortest heat dissipation time of the wax pattern was achieved when the surface roughness of the cooling channel is Sz 71.9 µm, with a corresponding coolant contact area of 3232 mm^2^. The heat dissipation time of the wax pattern increases when the coolant contact area is greater than 3232 mm^2^. While moderate roughness enhances turbulence and improves convective heat transfer, excessive roughness (beyond 71.9 µm) can cause flow separation, stagnation zones, and eddies. These disruptions can trap coolant in certain regions, reducing its ability to carry heat away efficiently. Therefore, the cooling effect was reduced when the coolant contact area was greater than 3232 mm^2^ [25]. Figure 12 shows the graphic depiction of the flow characteristics of cooling channels with surface roughnesses of Sz 1.9 µm, Sz 26.9 µm, Sz 71.9 µm, and Sz 105 µm. The Dye Tracing Method was used to inject a colorant into a fluid and observe the movement path of the dye to understand the flow pattern of the fluid. Turbulent flow [26] features chaotic and unpredictable fluid movement. This type of flow causes erratic fluctuations in velocity and pressure, creating a disordered, swirling motion. Turbulent flow enhances the mixing and dispersion of particles, heat, and momentum, but it also results in increased friction and pressure loss compared to laminar flow. Understanding turbulent flow is crucial for applications such as aircraft design, chemical reactors, and weather prediction.

**Figure 13:**
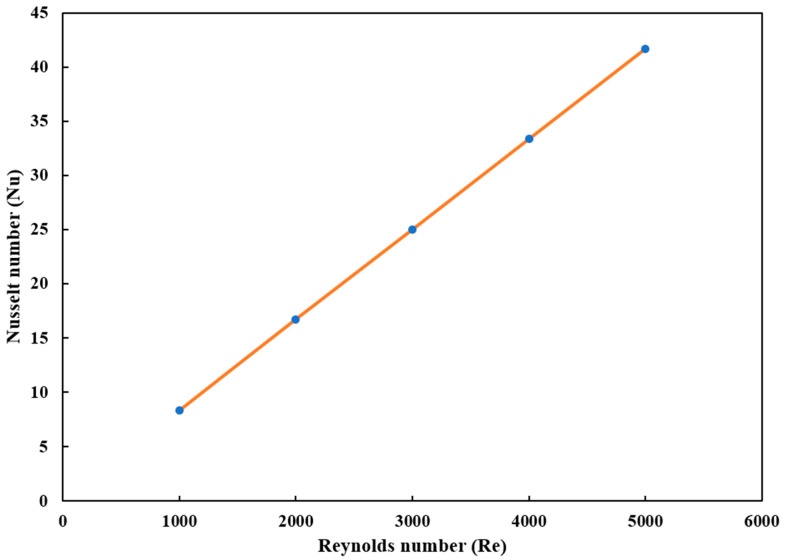
Correlation between the Nusselt and Reynolds numbers in this experiment.

The relationship between the Nusselt number (Nu) and the Reynolds number (Re) was analyzed to evaluate the effect of flow rate on heat transfer efficiency. Figure 13, the plot of Nu vs. Re demonstrates a linear increase in heat transfer efficiency with increasing flow rate, consistent with enhanced turbulence and improved convective heat transfer at higher Reynolds numbers. The effect of flow rate on heat transfer was analyzed by establishing a correlation between the Nusselt number (Nu) and the Reynolds number (Re). Using the Dittus–Boelter equation for turbulent flow,

Nu = 0.023Re^0.8^Pr^0.4^ (2)

**Figure 14:**
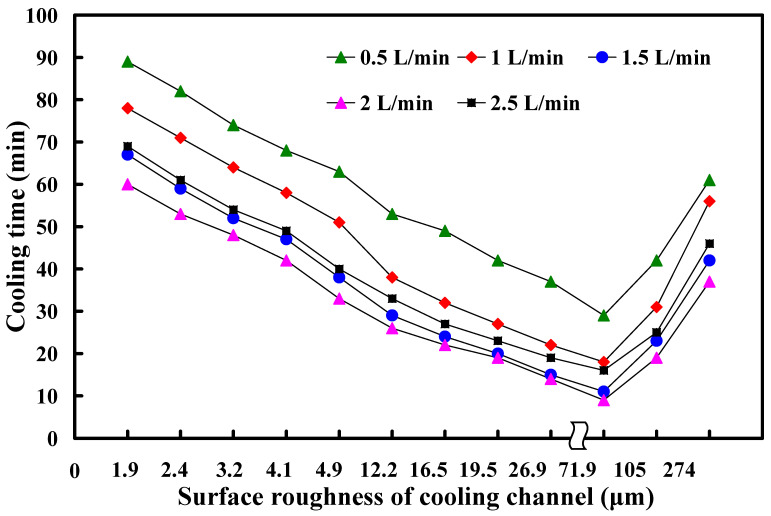
Relation between coolant flow rate and cooling time of the wax pattern.

To analyze the relation between coolant flow rate and cooling time of the wax pattern, this study employs a throttle valve in conjunction with a flow meter to regulate the relative flow rate. The flow rate parameters are set at intervals of 0.5 L/min, specifically at 0.5 L/min, 1.0 L/min, 1.5 L/min,2.0 L/min, and 2.5 L/min. Results are shown in Table 4. Figure 14 shows the relation between the coolant flow rate and heat dissipation time of the wax pattern. The findings showed that when the coolant flow rate ranges from 0.5 to 2 L/min, the heat dissipation time of the wax pattern decreases as the coolant flow rate increases. However, when the coolant flow rate increases to 2.5 L/min, the cooling efficiency does not increase but decreases. The main reason is that the coolant flow rate is too high and reduces the contact time between the coolant and the cooling waterway surface. This study found that a coolant flow rate of 2 L/min is the optimal coolant flow rate.

**Figure 15:**
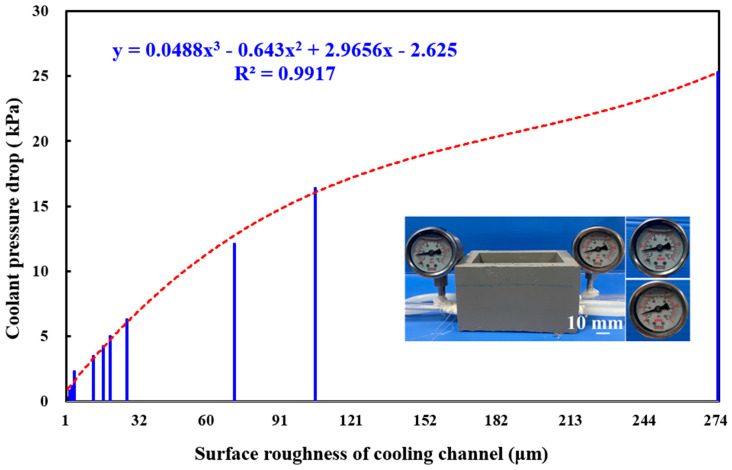
Coolant pressure drop between the inlet and outlet in cooling channels with varying surface roughnesses.

To investigate the pressure drop of coolant between the inlet and outlet in a series of cooling channels with varying surface roughnesses, this study selected 12 cooling channels with different surface roughness levels for experimentation. The surface roughness of 1.9 µm represents the original surface roughness of the cooling channels. Two materials were selected for machining the test specimens: aluminum rods were embossed on a lathe to produce high-surface-roughness channels, whereas high-speed steel rods were machined with a fiber laser to fabricate low-to-medium-surface-roughness channels. In total, 11 different surface roughness levels, ranging from low to high, were applied to the cooling channel surfaces. Surface roughness levels of 2.4 µm, 3.2 µm, 4.1 µm, 4.9 µm, 12.2 µm, 16.5 µm, 19.5 µm, and 26.9 µm were produced by laser machining the steel rods, while roughness levels of 71.9 µm, 105 µm, and 274 µm were achieved by embossing the aluminum rods on a lathe. Figure 15 illustrates the coolant pressure drop between the inlet and outlet for cooling channels with different surface roughness levels. The results indicate that the pressure drop values for channels with surface roughness levels of 1.9 µm, 2.4 µm, 3.2 µm, 4.1 µm, 4.9 µm, 12.2 µm, 16.5 µm, 19.5 µm, 26.9 µm, 71.9 µm, 105 µm, and 274 µm are approximately 0.29 kPa, 0.78 kPa, 1.18 kPa, 1.67 kPa, 2.26 kPa, 3.43 kPa, 4.22 kPa, 5 kPa, 6.28 kPa, 12.06 kPa, 16.38 kPa, and 25.3 kPa, respectively. Two key phenomena were observed. As the surface roughness of the cooling channel increases, the coolant pressure drop also increases. This trend is evident from the upward slope of the curve, indicating that higher surface roughness leads to greater resistance to coolant flow, resulting in higher pressure drops. Additionally, the coolant pressure drop (y) can be accurately predicted based on the surface roughness (x) of the cooling channels. The relationship follows the equation y = 0.0488x^3^ − 0.643x^2^ + 2.9656x − 2.625, with a high coefficient of determination of about 0.9917, indicating a strong fit between the model and the observed data.

In this work, the surface textures in the cooling channel surface were obtained by laser processing and embossing machining. Electrode discharge machining [27] can also create surface textures in the cooling channel surface because it provides exceptional precision for creating detailed microstructures, making it ideal for intricate designs. It handles materials, such as hardened steel and titanium, which are hard to machine with classic approaches. EDM produces superior surface finishes, crucial for microstructures where surface quality impacts performance. The process avoids direct contact between the tool and the workpiece, preventing mechanical stresses and distortions. Additionally, EDM achieves complex shapes and fine details that are hard to produce with conventional machining techniques. In addition, the future of rapid tooling involves the development of materials with enhanced properties, such as increased strength and thermal resistance [28], which will broaden its applications. Integrating digital technologies like the internet of things [29,30] and artificial intelligence [31] simplifies the design and production stages. Hybrid methods that combine additive and subtractive manufacturing offer greater precision and efficiency. Automation and robotics [32,33] reduce the need for human intervention, enhancing accuracy and consistency. Emphasis on sustainability leads to eco-friendly materials and processes, while advancements in software and collaborative platforms improve design, simulation, and innovation.

## 4. Conclusions

The study explores the development of aluminum-filled epoxy resin composites for rapid tooling in injection molding, highlighting the importance of optimizing mold design with cooling channels to enhance heat dissipation and reduce cooling time in wax injection molding processes. Results highlight the substantial effect of optimizing cooling channel surface roughness on improving heat dissipation and shortening cooling time in wax injection molding. By carefully designing and evaluating cooling channel parameters, the rapid tooling process can be significantly improved to deliver substantial benefits in terms of production efficiency and sustainability. The key conclusions from this research are:

1. The results of this study have significant contribution to cooling channel manufacturing process in the mold or die industry. Enhancing the surface roughness of the cooling channels in rapid molds significantly shortens the cooling times for wax patterns. Compared to the original smooth cooling channels, the optimal surface roughness of 71.9 µm improved cooling efficiency by approximately 81% translating to a time savings of around 12 s per molded product. This holds considerable significance for the investment casting industry, where the mass production of wax patterns is a common practice.
2. The relationship between cooling time and surface roughness can be accurately described by the obtained empirical equations. For parallel cooling channel configurations, the time duration required to cool (y) can be predicted using the equation y = 0.2015x^3^ − 3.2829x^2^ + 6.4581x + 76.615. For series cooling channels, the trend equation is y = 0.2022x^3^ − 3.2993x^2^ + 6.5816x + 79.457.
3. The pressure drop between the inlet and outlet of the cooling channels can be accurately predicted based on the surface roughness using the proposed equation, which yields a coefficient of determination of approximately 0.9917, indicating a high degree of correlation. This demonstrates the robustness and reliability of the model in characterizing fluid dynamics within the system.
4. The improved cooling performance is attributed to the enhanced turbulence and heat transfer characteristics induced by the increased surface roughness. This aligns with the principles of green manufacturing, reducing energy consumption and environmental impact in wax injection molding process. By providing a comprehensive analysis of the underlying mechanisms and practical implications, this study offers valuable insights to enhance the productivity, sustainability, and competitiveness of the investment casting industry through optimized rapid tooling solutions.

## Figures and Tables

**Table 1:** Key parameters affecting cooling performance.

Parameter	Value
Wax volume	128 cm^3^
Wax mass	115.2 g (0.1152 kg)
Initial temperature	85 °C
Final temperature	30 °C
Temperature change (ΔT)	55 K
Specific heat capacity (c)	2400 J/kg·K
Total heat removed (Q)	15.2 kJ
Thermal conductivity of wax	0.211 W/m·K
Mold thickness	4 cm
Epoxy-to-aluminum ratio	4:6

**Table 2:** Cooling times for parallel cooling channels with different surface roughnesses.

S. No.	Sz (µm)	Time (min)—Trial 1	Time (min)—Trial 2	Time (min)—Trial 3	Time (min)—Trial 4	Time (min)—Trial 5	Mean Time (min)
(i)	1.9	81	80	82	79	81	80.6
(ii)	2.4	78	77	79	78	77	77.8
(iii)	3.2	70	71	69	70	71	70.2
(iv)	4.1	65	64	66	65	64	64.8
(v)	4.9	55	54	56	55	55	55.0
(vi)	12.2	40	39	41	40	39	39.8
(vii)	16.5	30	31	29	30	30	30.0
(viii)	19.5	25	24	26	25	25	25.0
(ix)	26.9	20	19	21	20	20	20.0
(x)	71.9	15	16	14	15	15	15.0
(xi)	105	20	21	19	20	20	20.0
(xii)	274	30	31	29	30	30	30.0

**Table 3:** Cooling times for series cooling channels with different surface roughnesses.

S. No.	Sz (µm)	Time (min)—Trial 1	Time (min)—Trial 2	Time (min)—Trial 3	Time (min)—Trial 4	Time (min)—Trial 5	Mean Time (min)
(i)	1.9	85	84	86	85	84	84.8
(ii)	2.4	78	77	79	78	78	78.0
(iii)	3.2	72	71	73	72	72	72.0
(iv)	4.1	67	66	68	67	67	67.0
(v)	4.9	59	58	60	59	59	59.0
(vi)	12.2	41	40	42	41	41	41.0
(vii)	16.5	33	32	34	33	33	33.0
(viii)	19.5	27	26	28	27	27	27.0
(ix)	26.9	23	22	24	23	23	23.0
(x)	71.9	16	17	15	16	16	16.0
(xi)	105	20	21	19	20	20	20.0
(xii)	274	34	33	35	34	34	34.0

**Table 4:** Cooling time data for all coolant flow rates at different surface roughness values.

Surface Roughness (µm)	0.5 L/min	1.0 L/min	1.5 L/min	2.0 L/min	2.5 L/min
1.9	89	78	67	60	69
2.4	82	71	59	53	61
3.2	74	64	52	48	54
4.1	68	58	47	42	49
4.9	63	51	38	33	40
12.2	53	38	29	26	33
16.5	49	32	24	22	27
19.5	42	27	20	19	23
26.9	37	22	15	14	19
71.9	29	18	11	9	16
105	42	31	23	19	25
274	61	56	42	37	46

## Data Availability

The original contributions presented in this study are included in the article. Further inquiries can be directed to the corresponding authors.
